# Porcine reproductive and respiratory syndrome virus 2 in Europe: neither wild nor tamed

**DOI:** 10.1186/s40813-025-00478-x

**Published:** 2025-12-08

**Authors:** Giovanni Franzo, Claudia Maria Tucciarone, Matteo Legnardi, Irene Melegari, Shadia Berjaoui, Gardenia Gatta, Francesca Poletto, Roberto Valente, Debora Marcone, Alessio Lorusso, Michele Drigo, Umberto Molini

**Affiliations:** 1 Department of Animal Medicine, Production and Health, University of Padova, 35020 Legnaro, PD Italy; 2 Istituto Zooprofilattico Sperimentale dell’Abruzzo e del Molise, 64100 Teramo, Italy; 3 Azienda Sanitaria Locale 02, Lanciano Vasto Chieti, Via dei Vestini, 66100 Chieti, Italy

**Keywords:** PRRSV-2, Vaccine, Epidemiology, Europe, Dynamics, Phylogeograhy

## Abstract

**Background:**

Porcine reproductive and respiratory syndrome virus (PRRSV) exhibits a marked geographical clustering, with PRRSV-2 being predominantly found in North America and Asia. Its presence in Europe remains extremely limited and mainly represented by vaccine-like strains, belonging to sublineage 5.1. While the use of this vaccine is possible and applied in Northern Europe, its detection in Southern European countries has been largely anecdotal.

**Results:**

In the present study, we report the first confirmed case of PRRSV-2 detection in Italy, identifying two independent introduction events with no clear epidemiological link. A deeper evaluation of the overall epidemiological end evolutionary patterns of MLV-derived strains was performed to elucidate the present study findings. The phylogenetic and molecular analyses revealed significant genetic variability among vaccine-derived strains, with pairwise genetic distances exceeding 8% and an estimated evolutionary rate of ~10^− 3^ substitutions/site/year, comparable to field strains. These findings suggested ongoing viral evolution and persistent circulation since the vaccine introduction. Signatures of diversifying selection, particularly in ORF5, indicated adaptation to different host populations or immune environments. Moreover, phylogeographic analysis supported multiple independent introduction events of independently evolved strains rather than a single introduction followed by local evolution.

**Conclusions:**

Contrary to expectations, PRRSV-2 vaccine-like strains exhibited transmission dynamics comparable to field strains. Their divergence, potential for adaptation, and reversion to virulence raise concerns about their long-term epidemiological and clinical impact. Given the scarcity of PRRSV-2 field strain reports in Europe, further surveillance and sequencing efforts are crucial to assess its true prevalence, evolutionary potential, and implications for swine health.

**Supplementary information:**

The online version contains supplementary material available at 10.1186/s40813-025-00478-x.

## Background

Porcine reproductive and respiratory syndrome virus (PRRSV) was initially reported in the U.S. in 1987 and almost simultaneously in Europe and around the world, rapidly becoming the most impactful infectious disease affecting pig farming worldwide. It has been responsible for huge economic losses, both due to direct impacts on pig health and productivity and costs associated with its control [1].

The virus can infect pigs of all ages; however, its clinical manifestations are particularly severe in pregnant sows—especially during the last trimester—and young piglets. Infected sows may experience premature farrowing, resulting in stillbirths or the delivery of partially autolyzed or mummified fetuses. Young piglets frequently exhibit symptoms such as fever, severe dyspnea, anorexia, lethargy, decreased growth performances and mortality, whereas older pigs generally present with milder clinical signs. Moreover, PRRSV is a key etiological agent in the pathogenesis of complex syndromes, notably contributing to the development of respiratory disease complex in young piglets, where secondary infections often exacerbate disease severity [2–4]. Remarkably more severe disease have been observed for highly pathogenic strains, like Lena, AUT15-33, Rosalia, whose emergence has been reported worldwide, although the genetic and biological determinants of such virulence increase are still largely unknown [3].

Despite the largely overlapping history, biology, epidemiological pattern and clinicals signs, the porcine reproductive and respiratory syndrome (PRRS) is caused by two distinct viral species, *Betaarterivirus europensis* (commonly known as Porcine reproductive and respiratory syndrome virus 1; PRRSV-1) and *Betaarterivirus americense* (commonly known as Porcine reproductive and respiratory syndrome virus 2; PRRSV-2, or American type), both belonging to the genus *Betaarterivirus*, family *Arteriviridae* (https://ictv.global/taxonomy). Such classification reflects the substantial genetic divergence between PRRSV-1 and PRRSV-2, as they share only 55–65% nucleotide identity across their genomes. Additionally, this distinction aligns with a consistent geographical distribution, suggesting a long-term evolutionary divergence that has been largely maintained over time despite extensive global trade. PRRSV-1 was historically referred to as the European type, while PRRSV-2 was known as the North American type, although it is now the predominant variant also in Asia. Furthermore, both species exhibit high within-species genetic variability, driven by the high mutation and recombination rates characteristic of RNA viruses [5, 6].

PRRSV possesses a single-stranded, positive-sense RNA genome approximately 15 kb in length [(ss(+) RNA)], encoding both structural and non-structural proteins. Approximately 75% of the PRRSV genome is occupied by ORF1a and ORF1b, located at the 5’-terminus. These encode two large polyproteins (pp1a and pp1ab), which undergo autocatalytic cleavage to generate non-structural proteins (Nsps) that regulate viral replication and pathogenesis. The remaining 25% of the genome, located at the 3’-terminus, encodes eight structural proteins, including the envelope protein (E), glycoproteins GP2 to GP5, membrane protein (M), and nucleocapsid protein (N). Among those the GP5 and N, encoded by ORF5 and 7, are by far the most commonly studied and sequenced, both because their biological role and immunological relevance, and because of their variability, which made them optimal targets for molecular epidemiology studies [7, 8].

While biosecurity measures and farm management are the cornerstones of PRRSV control and eventual eradication, vaccination is commonly employed to mitigate clinical signs and reduce viral circulation, particularly with modified live vaccines (MLV). However, the high genetic variability of PRRSV often hinders cross-protection, thereby diminishing the effectiveness of this approach [9, 10]. Moreover, the use of live vaccines is not fully devoid of risks, including reversion to virulence [11–13]. PRRSV-2 strains have been sporadically reported in Europe, with the first documented introduction occurring in 1996, primarily due to the use of MLVs [14]. These vaccine-derived strains subsequently spread among farms, sometimes in association with clinical manifestations attributable to reversion to virulence. Subsequent reports have demonstrated the circulation of PRRSV-2 strains in several European countries, where many of the strains are genetically related to the Ingelvac PRRSV MLV [6, 15, 16]. However, their genetic divergence (~5%) challenges a definitive attribution of a vaccine origin beyond any reasonable doubt or, at the very least, the comprehension of the underlying pattern of PRRSV-2 circulation in Europe. Non-vaccine strains have also been detected, but they remain substantially sporadic and isolate reports [15, 17].

In Italy, despite anecdotal rumours and a widely shared consensus among field veterinarians regarding the presence of PRRSV-2 strains, no official reports or publicly available sequences have been published to characterize these strains. The present study, building on the first confirmed detections of PRRSV-2 MLV-like strains in Italy, contextualizes them within the broader international scenario. It assesses their epidemiology and evolutionary dynamics on a wider geographical and temporal scale, highlighting the importance of this topic, the existing knowledge gaps, and the urgent need for more in-depth diagnostic investigations.

## Material and methods

### Italian samples

Samples included in the study were independently delivered to the laboratory of infectious disease of the Dept. of Animal Medicine, Production and Health (MAPS, Legnaro, PD, Italy) and the Istituto Zooprofilattico Sperimentale dell’Abruzzo e del Molise “Giuseppe Caporale” (IZSAM, Teramo, Italy), for routine diagnostic purpose.

The two samples delivered to the MAPS laboratories originated from a nursery (site 2) farm located in Northern-eastern Italy in October 2024 and were collected for routine surveillance purposes. The farm received animals from another Italian sow farm (site 1), which was part of an integrated flow and no contact or epidemiological links with foreign countries could be identified. The farm had a history of PRRSV-1 circulation.

The samples submitted to IZSAM originated from a farm located in Central Italy in November 2024. In this second farm, animals had been imported from Denmark for the finishing phase. Diagnosis was initially performed at both laboratories using the VetMAX™ PRRSV EU & NA 2.0 Kit (Applied Biosystems™, Waltham, MA, USA) and then ORF5 and ORF7 portions of positive samples were amplified at MAPS laboratories using the primer described by Oleksiewicz et al. 1998 [18], and Sanger sequenced at Macrogen Europe using the same primers. Chromatograms were evaluated with FinchTV (http://www.geospiza.com) and consensus sequences were obtained using CromasPro (CromasPro Version 1.5).

### Reference datasets preparations

To focus the analysis on strains closely related to the Italian ones, a BLAST analysis was initially performed to identify and retrieve ORF5 and ORF7 sequences with a percentage identity higher than 90% and gene coverage exceeding 95%.

Additionally, to obtain a comprehensive representation of PRRSV-2 genetic variability, a random subset of 2,000 sequences was selected and downloaded from NCBI Virus (https://www.ncbi.nlm.nih.gov/labs/virus/vssi/#/). When available, metadata on host, country, and collection date were annotated. Previously published European PRRSV-2 [15, 16] sequences and vaccine-derived sequences were also retrieved and included in the dataset.

All sequences were aligned using the MAFFT [19] method implemented in TranslatorX [20], accounting for the coding nature of ORF5 and ORF7. The alignment quality was assessed through visual inspection, and sequences exhibiting poor quality, frameshift mutations, or premature stop codons were excluded from further analysis. Strains were classified as European or Non-European depending on the country of origin.

### Sequence analysis

Preliminary phylogenetic trees for ORF5 and ORF7 were generated for classification purposes using IQ-TREE [21], selecting the substitution model with the lowest Akaike Information Criterion (AIC), as determined by the same software. The reliability of the inferred clusters was assessed by calculating the SH-aLRT test [22].

Strains clustering with Ingelvac PRRSV MLV were identified, and independent datasets were generated. To further confirm and strengthen the association with the vaccine, vaccine-related sequences were iteratively aligned with vaccine reference sequences, retaining only those with a percentage identity higher than 95%. Further confirmation was obtained using the automatic PRRSV-2 lineage and sublineage classification tool, as described by VanderWaal et al. [23].

Pairwise genetic distances were calculated among vaccine-like strains circulating in Europe, outside Europe, and between these two groups. A phylogenetic analysis was also performed on vaccine-like strains, following the methodology described previously. An ancestral state reconstruction, categorizing sequences as either European or Non-European, was conducted using the *ape* [24] package in R [25] based on these phylogenetic trees. This analysis aimed to infer the most likely geographic location of ancestral nodes.

### Evaluation of vaccine-like evolution and action of selective pressures

The selected datasets were analyzed to infer various population parameters, including the time to the most recent common ancestor (tMRCA), evolutionary rate, and viral population dynamics, using the Bayesian serial coalescent approach implemented in BEAST 1.10 [26]. The nucleotide substitution model was chosen based on the Bayesian Information Criterion (BIC), as calculated using JModelTest2 [27]. To determine the most appropriate molecular clock model, the marginal likelihood estimation was assessed using path-sampling and stepping-stone methods, following the recommendations of Baele et al. [28].

The non-parametric Bayesian Skygrid model was applied to account for viral population changes over time, estimating relative genetic diversity (effective population size × generation time; Ne × τ) [29]. Additionally, a discrete trait analysis (DTA) was performed as described by Lemey et al. [30], using an asymmetric migration model with Bayesian stochastic search variable selection (BSSVS) to estimate the strain ancestral locations (categorized as European and Non-European).

Two independent Markov Chain Monte Carlo (MCMC) runs, each consisting of 200 million generations, were conducted on ORF5 and ORF7 PRRSV-2 vaccine-like strains. Convergence and mixing were evaluated using Tracer 1.7 [31], and results were accepted only if the effective sample size (ESS) was greater than 200. Parameter estimates were summarized as mean values with 95% highest posterior density (HPD) intervals. Maximum clade credibility (MCC) trees were generated and annotated using TreeAnnotator (BEAST 1.10 package). Additional summary statistics and graphical outputs were generated using custom R scripts.

The occurrence of gene-wide episodic diversifying selection was assessed at the gene level using BUSTED [32], which accounts for synonymous rate variation and error protection (BUSTED-ES). Additionally, selection at individual sites was evaluated and reported as evidence ratios (ERs), with an ER > 10 considered indicative of diversifying selection acting at that site.

Based on the assumption that evolutionary patterns are influenced by environmental determinants, a contrast-MEME analysis was conducted, as implemented in HyPhy [33]. This approach allowed for the evaluation of differences in episodic diversifying selection acting on distinct sets of branches. Specifically, branches leading to European strains were compared to non-European ones. Ancestral lineages were assigned to one of the two categories using a parsimony-based approach. The statistical significance threshold was set at *p* < 0.05.

## Results

Two samples from Northern Italy and one from Central Italy tested PRRSV-2 positive. The complete ORF7 sequence was successfully obtained for all samples, while ORF5 characterization was possible only for the northern Italian samples. Sequences have been submitted to GenBank with the accession number PV188150 - PV188154)

The final reference database used for comparison included 2841 ORF5 sequences and 2001 ORF7 sequences, of which 1749 and 311 were classified within the vaccine-like cluster, namely variant 5A.1 (Ingelvac PRRS MLV; Boehringer Ingelheim Animal Health, Duluth, GA). With few exceptions, nearly all European sequences belonged to this group (Supplementary Figure 1).

**Fig. 1:**
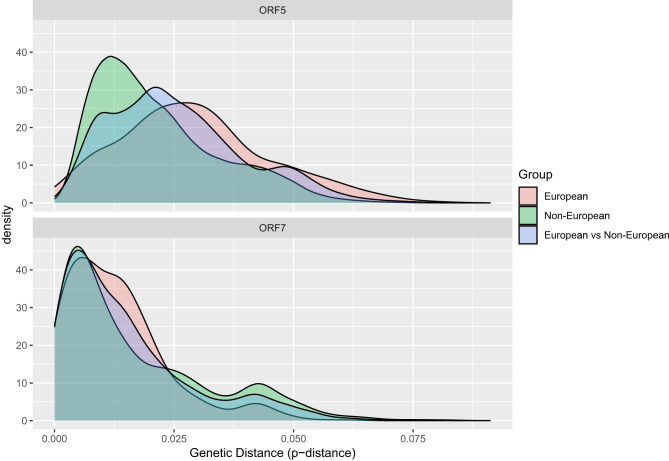
Density plot of pairwise genetic distances (p-distance) for ORF5 and ORF7 sequences of PRRSV-2. The plots compare intra-European (pink), intra-non-European (green), and intercontinental (European vs. Non-European, blue) genetic distances. The x-axis represents the genetic distance, while the y-axis shows the value density distribution

The pairwise genetic distance comparison showed a significant genetic distance among strains included in this group, both for ORF5 and ORF7 (Fig. 1). Up to ~9% genetic distance was calculated within both datasets, possibly suggesting a divergent evolution of strains, which could have differentiated in different direction from the vaccine. Despite there was a substantial overlapping among considered groups, a tendency toward a slightly higher variability was observed among European strains, especially in the ORF5.

**Fig. 2:**
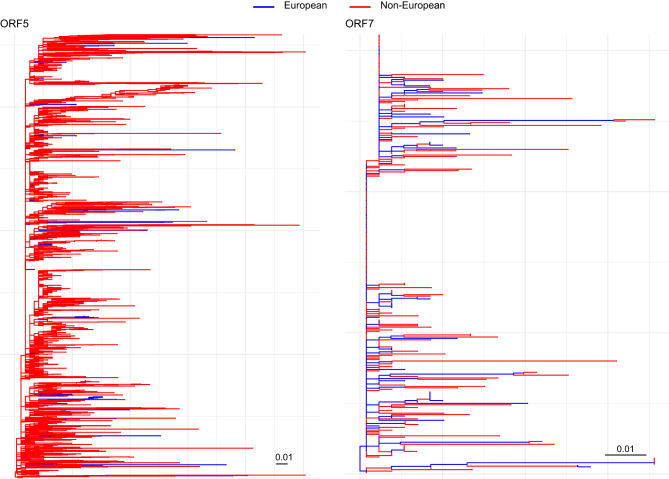
Maximum likelihood (ml) phylogenetic trees for ORF5 (left) and ORF7 (right) sequences, reconstructed to assess the evolutionary relationships of European (blue) and non-European (red) strains

The phylogenetic analysis of the Vaccine-like dataset revealed that European strains, while forming small clusters in some instances, were largely interspersed with Non-European strains across both datasets. Additionally, the maximum likelihood (ML)-based reconstruction of ancestral states estimated multiple state transitions throughout the evolutionary history of the virus (Fig. 2), suggesting frequent exchanges between European and Non-European locations. Newly identified Italian strains were part of 2 independent clades, suggesting independent introduction events (Supplementary figure 1)

**Fig. 3:**
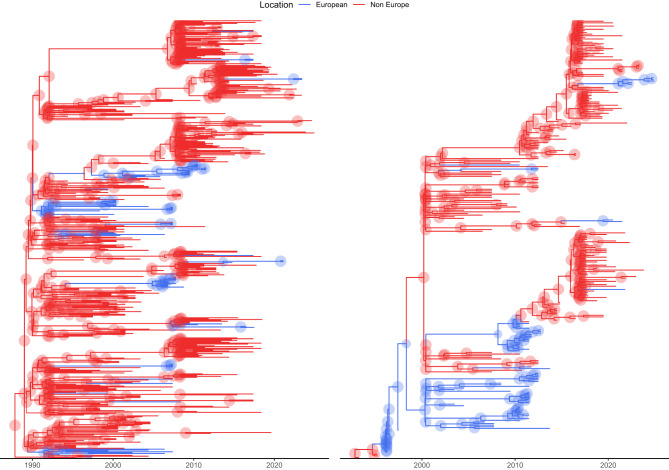
Time-scaled Bayesian phylogenetic trees for ORF5 (left) and ORF7 (right), inferred using beast. Branch colors represent the inferred ancestral location, with European strains in red and non-European strains in blue. Shaded circles indicate the posterior probability of ancestral state reconstructions at each node

The DTA performed with the serial coalescent approach provided similar evidence, although differences in tree structure were present between the two analysis (Fig. 3).

The same analysis inferred a tMRCA in 1991.12 [95HPD: 1988.01–1994.18] and 1991.16 [95HPD: 1984.78–1993.45] for the ORF5 and ORF7 dataset, and a corresponding evolutionary rate of 3.15 × 10^−3^ [95HPD: 2.44 × 10^−3^ − 3.97 × 10^−3^] and 2.99 × 10^−3^ [95HPD: 1.68 × 10^−3^ −4.33 × 10^−3^] substitutions/site/year.

The analysis of selective pressures identified evidence of episodic diversifying selection at the gene level in both ORF5 and ORF7 within the vaccine-like group. Notably, sites in ORF5 exhibiting an evidence ratio (ER) > 10 included positions 3, 4, 5, 11, 12, 13, 28, 32, 33, 34, 47, 57, 58, 79, 80, 81, 106, 117, 182, 185, 193, 194, and 196. Furthermore, differences in selective pressures acting on strains circulating in European and non-European countries were detected at positions 3, 11, 13, 29, 58, 117, and 196, suggesting distinct evolutionary constraints across geographical regions. In the ORF7, BUSTED reported evidence of selection at sites 9, 11, 33,47,49,54 and 122, while no differences among geographic groups were detected with contrast-MEME.

**Fig. 4:**
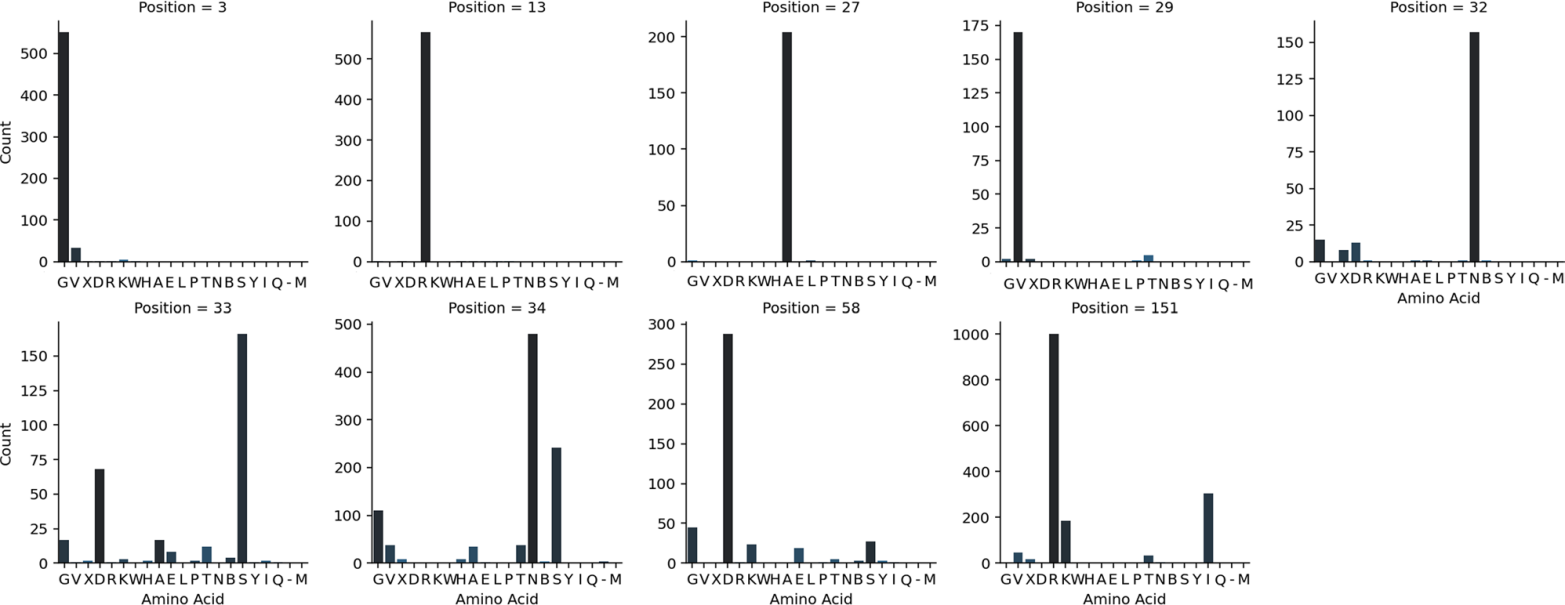
Amino acid variability at key positions in PRRSV-2 vaccine-like ORF5 sequences compared to vaccine reference. Bar plots display the frequency of amino acid variants at selected positions where more than 10% of sequences differed from the ingelvac PRRSV MLV reference strain. The x-axis represents different amino acid residues, while the y-axis indicates the number of sequences exhibiting the corresponding residue at each position

The comparison of vaccine-like sequences with the Ingelvac PRRSV MLV reference revealed the presence of multiple mutations. To minimize the inclusion of low-fitness, transitory mutations, only positions where more than 10% of the sequences differed from the vaccine reference were considered. Notably, in ORF5 positions 3, 13, 27, 29, 32, 33, 34, 58, and 151 were identified, with positions 32–151 exhibiting the highest amino acid variability (Fig. 4).

**Fig. 5:**
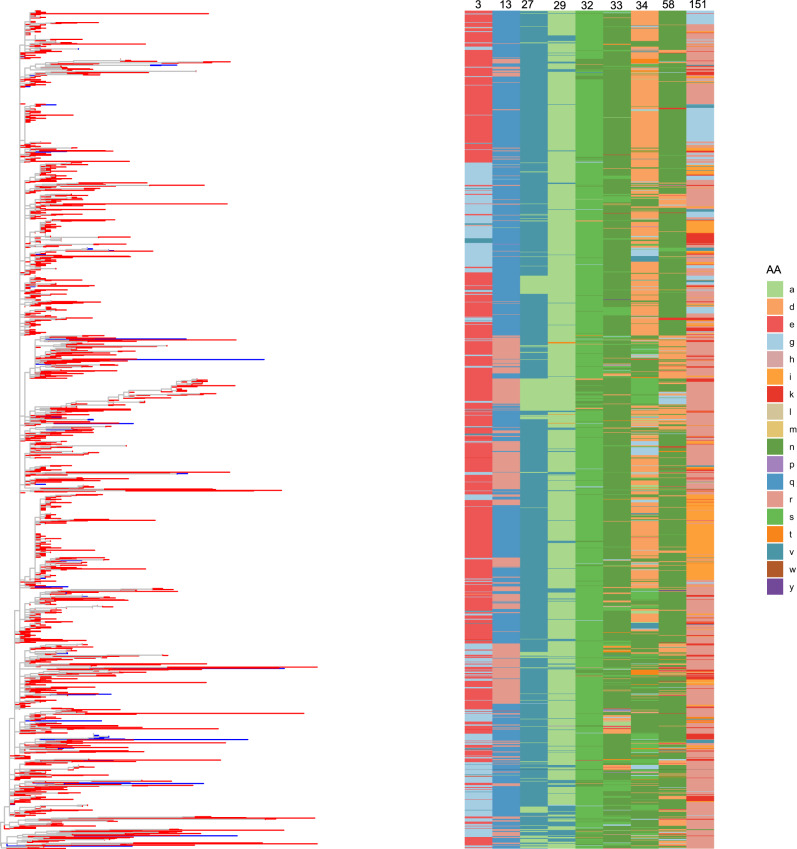
Maximum likelihood phylogenetic tree (left) and amino acid variability heatmap (right) for PRRSV-2 strains. The phylogenetic tree illustrates the evolutionary relationships among ORF5 sequences, terminal branch are coloured based on the collection region (i.e. Europe = blue; outside Europe = red). The heatmap displays amino acid variations at key positions (columns) across different sequences (rows), with colours representing different amino acid residues

Although clear topological clustering was observed, with closely related strains exhibiting similar amino acid profiles, identical amino acid substitutions were also present in phylogenetically distant branches (Fig. 5). This pattern suggests that specific variations have independently emerged multiple times throughout the evolutionary history of PRRSV MLV-like strains. Additionally, mutations occurring at different positions were not necessarily linked, as diverse combinations of amino acid substitutions were observed across strains. Only position 38 was featured by a significant variability compared to the reference vaccine in the ORF7.

## Discussion

The two viral species responsible for PRRSV exhibit a marked geographical clustering. Despite the presence of the “American” type, PRRSV-2, in Europe, its occurrence remains extremely limited and, as confirmed by the present study, detected strains were closely related to Ingelvac PRRSV MLV and belonged to sublineage 5A.1, with extremely limited exceptions. The use of this vaccine dates back to 1994 in North America and was thereafter introduced in Europe two years later, where it is still currently authorized and available in several EU countries (https://medicines.health.europa.eu/veterinary). While vaccination with Ingelvac PRRSV MLV remains common in Northern European countries—particularly in Denmark—its use is less prevalent in Southern and Mediterranean regions and precise data on its administration are not available. Nonetheless, its circulation is anecdotally reported and widely recognized among veterinarians in these areas, often linked to the introduction of animals from Denmark. This appears to be the case for the Italian strain characterized in the present study. However, scientific literature on this topic remains scarce, as evidenced by this being the first confirmed report of PRRSV-2 detection in Italy. While routine diagnostic approaches may be considered adequate from a field perspective and for animal management, they are insufficient for identifying more complex epidemiological patterns. The introduction of animals from Denmark can easily explain the detection of a PRRSV-2 vaccine-like strain in the Central Italy farm under consideration. However, the same cannot be said for the Northern Italian farm, where no evidence of animal introduction from other countries or any other overt epidemiological link was identified, suggesting local viral circulation followed by introduction into the farm through pathways comparable to those of field strains.

Moreover, although the low viral titer of the Central Italy sample only allowed for the characterization of the ORF7 gene, it was sufficient to confidently assess its variability compared to strains identified in the same period in Northern Italy. This suggests independent introduction events from distinct sources with different evolutionary histories. Such findings are surprising, considering that little to no evolution is expected (or desirable) from a vaccine strain. Contrary to this expectation, the global analysis of MLV-like strains, including those circulating in Europe, revealed a markedly different picture. The pairwise genetic distance between these strains exceeded 8% for both ORFs, regardless of the geographic area considered. Given that the threshold for inclusion in the vaccine-like clade was set at a genetic distance of less than 5% from the reference vaccine, the presence of sequence pairs with significantly higher divergence suggests a substantial evolutionary capacity and a progressive divergence trend. Moreover, the estimated evolutionary rate for this clade was in the range of 10^− 3^ substitutions/site/year, comparable to that of field strains. The estimated time to the most recent common ancestor (tMRCA) closely aligned with the initial introduction of the vaccine in the U.S. and Europe, supporting the hypothesis of persistent circulation and ongoing evolution in the field since its introduction. It must be stressed that coalescent-based analyses are not well-suited for investigating vaccine strains, as the sampling time does not reflect the evolutionary time because they originate from the same vaccine batch and remain genetically unchanged during prolonged storage before administration in the field [34]. Nevertheless, while considering the aforementioned caveats, the estimated evolutionary rate and the presence of a certain temporal signal also contributed to support the occurrence of evolution of modified live vaccines. Comparably, in vaccine-associated sublineage reported in US, several well-supported small clusters were observed. These may reflect the transmission of vaccine-derived viruses in the field [1]. Consistent with this, evidence of episodic diversifying selection was detected at both the gene and site-specific levels, particularly in ORF5, including positions predicted to be exposed on the viral surface (data not shown). The variability in selective pressures acting on strains circulating in different environments strongly suggests sustained transmission in host populations with distinct immunological backgrounds, leading to differential selective constraints and evolutionary trajectories. Taken together, these pieces of evidence advocate that MLV-like strains share several epidemiological and evolutionary features with field strains. From a geographical perspective, the European and non-European countries were interspersed within the phylogenetic tree. Additionally, the discrete trait analysis further supports frequent state changes over time. Such a pattern is typically indicative of extensive viral migration and multiple introduction events. However, this evidence conflicts with what is observed for PRRSV-2 field strains. If such an intense viral flow were occurring, a similar phylogeographic pattern would be expected for field strains as well. On the contrary, only extremely limited reports of PRRSV-2 field strains have been documented to date [15]. Explaining this discrepancy solely based on epidemiological determinants seems unlikely, suggesting that additional factors may be influencing the spread and persistence of MLV-like strains in different regions.

An alternative and more parsimonious hypothesis would claim the occurrence of several, independent, evolution events originating from the same vaccine strain after administration, which could lead to convergent evolutive adaptation in different places, indirectly causing genetically related strains to evolve multiple times in different places. Initial studies investigating the early MLV- induced outbreaks occurred in Denmark highlighted several mutations pointing toward reversion to virulence, including parallel mutations occurred independently in different strains [35, 36], and described in North American isolates also [37]. The presence of these reversions in two different continents strongly suggests their role in both the attenuation of the vaccine virus and its subsequent reversion to virulence under natural selection pressure. The results of this study confirm and extend these initial findings, benefiting from a much larger dataset, particularly for ORF5.

A limited number of positions showed significant variability compared to the vaccine strain, indicating that despite the virus high mutation rate, non-synonymous mutations are tolerated or positively selected only within specific evolutionary pathways. However, within these constrained sites, multiple adaptive solutions were observed, with different amino acid substitutions becoming predominant—particularly in regions 32–58 and at position 151. In contrast, positions 3, 13, 27, and 29 displayed only a single dominant amino acid change. Notably, positions 13 and 151 were previously reported in the Danish outbreak as sites where mutations reverted toward the parental strain [14]. While this study confirms the importance of these positions, it also demonstrates that they are not strictly constrained, as alternative mutations were also viable. The evaluation of amino acid variability across the phylogenetic tree revealed that the same mutations, leading to multiple alternative amino acids, emerged independently several times across different branches and locations. This pattern proposes that, while the range of viable evolutionary pathways remains limited, it is still broad enough to generate diverse adaptive variants. Although the expected linkage among mutations due to their descent from a common ancestor was evident, variability in the amino acids at different positions was also observed. This highlights the evolutionary plasticity of PRRSV, even in strains derived from the MLV vaccine. An alternative explanation to independent *de novo* reversions is that the same strong selective pressure favored the selection of residual VR2332-like pathogenic virus which may have remained in the vaccine formulation [35]. If the vaccine virus was not fully purified during attenuation, small amounts of the original pathogenic strain could have persisted. In this scenario, selection might have primarily acted on a single site, while other parallel mutations could have arisen due to genetic linkage rather than independent adaptation [35]. A combination of both the presence of partially attenuated subpopulation followed by in vivo adaptation, leading to the observed heterogenicity of MLV derived strains, is also possible [38].

The analysis of evolutionary patterns and molecular epidemiology of PRRSV-2 in Europe reveals a far more heterogeneous and dynamic scenario than commonly perceived by field veterinarians, who often consider the detection of this viral type as a given fact with limited clinical or practical implications. Although constrained by data scarcity, the findings of this study suggest that, in several respects, MLV-like strains exhibit persistence and transmission patterns comparable to those of field strains, indicating a significant evolutionary potential. Since viral evolution generally favors more adapted and, consequently, potentially more virulent variants, it is plausible that clinically relevant forms may emerge—or may already be circulating misdiagnosed. The current lack of interest in more extensive diagnostics, sequencing, and reporting represents a major gap in our understanding of PRRSV-2 in Europe. This limitation prevents an objective assessment of its present impact on swine farming and its potential to become a more significant threat in the future.

## Electronic supplementary material

Below is the link to the electronic supplementary material.

Supplementary Material 1 Maximum likelihood (ML) phylogenetic trees for ORF5 (first page) and ORF7 (first page) sequences, reconstructed to assess the evolutionary relationships of European (blue) and Non-European (red) strains. Vaccine-related clade has been highlighted in yellow

## Data Availability

All obtained sequences have been made available in GenBank under the accession numbers PV188150 - PV188154.
