# Supplementary material for: Porcine reproductive and respiratory syndrome virus 2 in Europe: neither wild nor tamed
Source: Porcine Health Manag. 2025 Dec 8;11:62. doi: 10.1186/s40813-025-00478-x (PMC12752272; doi:10.1186/s40813-025-00478-x)

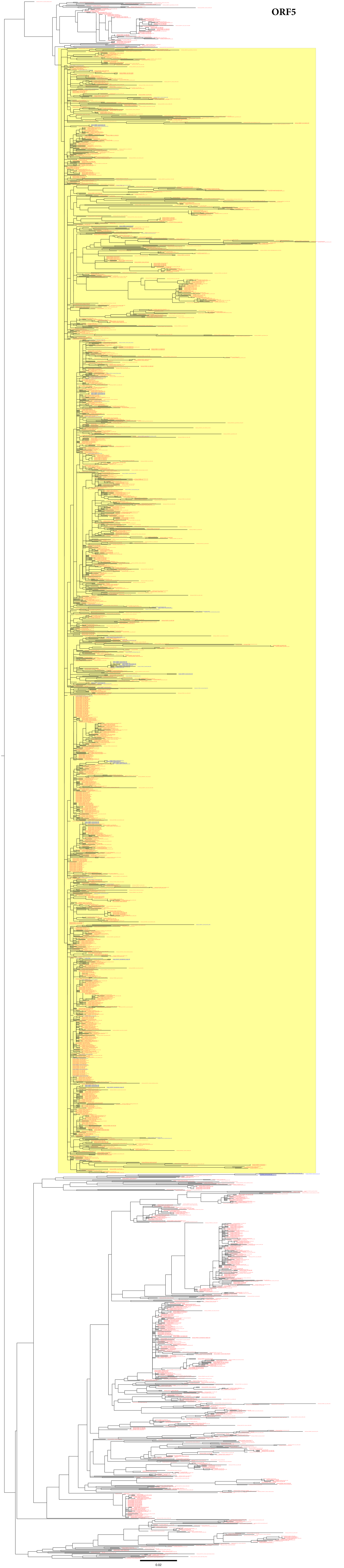

ORF5
0.02

ORF7

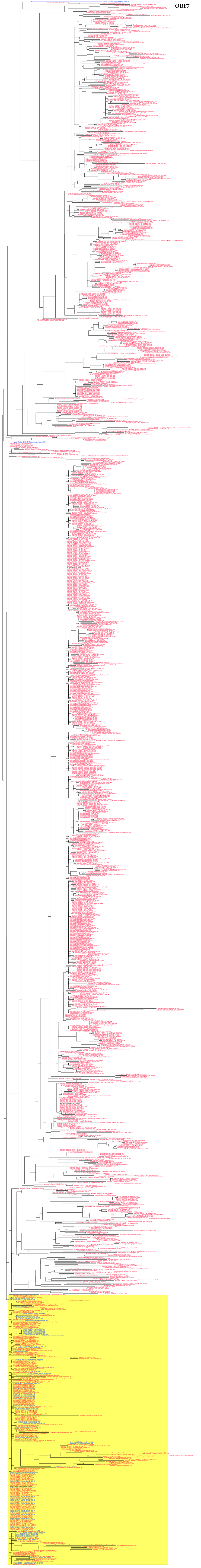

Supplement: Supplementary file 1 — Supplementary Material 1 Maximum likelihood (ML) phylogenetic trees for ORF5 (first page) and ORF7 (first page) sequences, reconstructed to assess the evolutionary relationships of European (blue) and Non-European (red) strains. Vaccine-related clade has been highlighted in yellow [file 40813_2025_478_MOESM1_ESM.pdf]
